# Deletion of FADD in Macrophages and Granulocytes Results in RIP3- and MyD88-Dependent Systemic Inflammation

**DOI:** 10.1371/journal.pone.0124391

**Published:** 2015-04-13

**Authors:** Suruchi N. Schock, Jennifer A. Young, Tina H. He, Yuefang Sun, Astar Winoto

**Affiliations:** Department of Molecular and Cell Biology and Cancer Research Laboratory, University of California, Berkeley, California, United States of America; Johns Hopkins School of Medicine, UNITED STATES

## Abstract

Myeloid cells, which include monocytes, macrophages, and granulocytes, are important innate immune cells, but the mechanism and downstream effect of their cell death on the immune system is not completely clear. Necroptosis is an alternate form of cell death that can be triggered when death receptor-mediated apoptosis is blocked, for example, in stimulated Fas-associated Death Domain (FADD) deficient cells. We report here that mice deficient for FADD in myeloid cells (mFADD^-/-^) exhibit systemic inflammation with elevated inflammatory cytokines and increased levels of myeloid and B cell populations while their dendritic and T cell numbers are normal. These phenotypes were abolished when RIP3 deficiency was introduced, suggesting that systemic inflammation is caused by RIP3-dependent necroptotic and/or inflammatory activity. We further found that loss of MyD88 can rescue the systemic inflammation observed in these mice. These phenotypes are surprisingly similar to that of dendritic cell (DC)-specific FADD deficient mice with the exception that DC numbers are normal in mFADD^-/-^ mice. Together these data support the notion that innate immune cells are constantly being stimulated through the MyD88-dependent pathway and aberrations in their cell death machinery can result in systemic effects on the immune system.

## Introduction

Dendritic cells (DCs), macrophages and monocytes are closely related cells derived from the same common myeloid progenitors [1,2]. They share common functions like antigen presentation, participation in T cell development and maintenance of gut immune system homeostasis. However, each also plays additional distinct roles in the immune system [3,4]. DCs are required for initiation of immunity; DC-less mice exhibit impaired innate immunity and diminished NK and CD8^+^ T cell responses to infection [5,6]. DCs also play an important role in peripheral T cell tolerance as mice with apoptosis-resistant DCs develop autoimmunity [7,8]. In contrast, loss of macrophages and monocytes has no overt effects on innate immunity but instead results in reduced Th1 adaptive immunity or defective wound healing [9,10].

Although regulation of cell death in macrophages and granulocytes is not fully understood, pyroptotic death has been reported to occur in macrophages infected with intracellular bacteria [11–13]. Pyroptosis is similar to necrotic death but is initiated by caspase-1 activation, resulting in the release of the inflammatory cytokines IL-1 and IL-18. However, a recent paper reported that the intracellular bacteria *Salmonella Typhimurium* [14] is capable of initiating another form of cell death termed necroptosis in macrophages. Necroptosis is necrotic death that is dependent on the activities of several genes, including the RIP1 death domain-containing kinase and its family member RIP3 [15–29]. Stimulation with apoptotic-inducing ligands, such as tumor necrosis factor (TNF), triggers necroptosis in apoptotic-resistant cells (e.g. cells deficient in Fas-associated death domain (FADD) or caspase-8) [23,28,30]. However, other stimuli apart from the TNF superfamily ligands can also induce necroptosis. For example, T cell receptor engagement in T cells lacking caspase-8 or FADD, the adapter protein for all the TNF-death receptor family members [31–33], activates necroptosis. Similarly, dendritic cells lacking FADD undergo necroptosis when their Toll-like receptors (TLRs) are stimulated [34]. Macrophages treated with zVAD-FMK, a general caspase inhibitor, and TLR ligands can also die through necroptosis [35,36]. In cases where TIR domain-containing adaptor inducing interferon-β (TRIF) acts as the adaptor molecule, TLR-induced necroptosis can be initiated by direct recruitment of RIP3 to the adapter protein TRIF. In contrast, necroptosis mediated by MyD88 is thought to proceed through a TNF-dependent mechanism [35,36].

In addition to its role in necroptosis induction, RIP3 has been recently reported to promote inflammation in a direct, necroptosis-independent fashion [37–40]. LPS stimulated macrophages can activate RIP3-dependent production of pro-inflammatory cytokines IL1 and IL18 upon SMAC mimetic induced IAP degradation [41]. In addition, LPS treatment of caspase-8 null DCs lead to increased inflammasome activation and IL1 secretion [42]. The pathway leading to IL1 production in DCs includes many of the same proteins that are important in necroptotic cell death, including RIP1, RIP3, FADD, and caspase-8 [38,39,42]. RIP3, in particular, can mediate activation of both caspase-1 and caspase-8 mediated inflammation [38,39]. However, the significance of FADD function in macrophages *in vivo* and the subsequent consequences on the immune system are not clear.

Recently, we have generated and analyzed DC-specific *FADD*-deficient (dcFADD^-/-^) mice and reported that these mice suffer from chronic inflammation with increased B cells, myeloid cells, macrophages and slightly elevated levels of TNF and *γ*-IFN [34]. RIP3 deficiency rescued these phenotypes, suggesting that *FADD*-deficient DCs undergo necroptosis *in vivo*. We demonstrated that DCs in gut-associated lymphoid tissues (GALT) are stimulated to undergo necroptosis in response to commensal bacteria [34]. Surprisingly, MyD88 in non-DC cells is also important for necroptosis-induced inflammation as complete loss of MyD88 but not DC-specific deletion can rescue dcFADD^-/-^ phenotypes. These data suggest that commensal bacteria may provide tonic signals through the MyD88 pathway in other innate immune cells. In the absence of these tonic signals, these cells are incapable of responding to the inflammatory contents released by necroptotic DCs and thus can no longer induce systemic inflammation. Given the similarity in functions between DCs and macrophages/myeloid cells, especially in the context of mucosal immunity [3], we investigated whether macrophages sensitive to necroptosis might also alter immune homeostasis in macrophage-specific *FADD*-deficient mice similar to dcFADD^-/-^ mice or if they will behave more like T-cell specific *FADD* knockout (tFADD^-/-^) mice with impairment isolated only to T cells [33]. Here, we report the generation and characterization of *FADD* ^*fl/fl*^ mice crossed to *Lysozyme-Cre (LysM-Cre)* transgenic mice, termed mFADD^-/-^ mice. Interestingly, mFADD^-/-^ mice show many similarities to that of dcFADD^-/-^ mice. These mice exhibit chronic inflammation with increased B cells and myeloid cells while T cell and DC numbers are normal in all lymphoid compartments examined. Unexpectedly, the numbers of macrophages and neutrophils are not decreased but are instead elevated. Loss of RIP3 rescued the mFADD^-/-^ phenotype, indicating that these phenotypes are due to RIP3-dependent necroptosis and/or inflammatory activity. We also found that systemic inflammation was abrogated following deletion of MyD88 in these mice. These data illustrate a dynamic interplay between macrophages and other innate cells while demonstrating the importance of MyD88 in maintaining immune system homeostasis.

## Materials and Methods

### Ethics Statement

All animals were handled in strict accordance with good animal practice as defined by the relevant national and/or local animal welfare bodies, and all animal work was approved by the UC Berkeley ACUC Animal Care and Use Committee.

### Mice

Mice were sacrificed using carbon dioxide (CO2) followed by cervical dislocation at 6–12 weeks of age unless otherwise noted. Littermates or sex- and age-matched mice were used as controls. mFADD^-/-^ mice were generated by crossing *LysM-Cre* transgenic mice with C57BL/6 *FADD* ^*flox/flox*^ mice generated previously in the lab [33]. *MyD88* ^*-/-*^mice (C57BL/6) were provided by Dr. Shizuo Akira [43] through Dr. Greg Barton, and *RIP3* ^-/-^ mice were from Xiaodong Wang [22]. Experimental mice were housed in the animal facility at the University of California, Berkeley.

### Macrophage Cell Enrichment

For collagenase digestion, spleens were cut into small pieces and digested with collagenase VIII at 37°C for 35 min, then incubated with 25 mM EDTA for 5 min at room temperature. Cells were dissociated and filtered through a 100 μm strainer. Alternatively, spleens were directly dissociated through a 100 μm strainer in 5ml of PBS. Red blood cell lysis with ACK lysis buffer was performed, and single cell suspensions were generated for downstream application.

For BMDM cultures, bone marrow was flushed from femurs and tibias using a needle and syringe, and single cell suspensions were filtered through 100 μm strainer. Red blood cell lysis was performed and cells were cultured in complete RPMI media (RPMI-1640 supplemented with 10% FCS, L-glutamine, penicillin-streptomycin, sodium pyruvate, 2-mercaptoethanol) and macrophage colony-stimulating factor (MCSF). Fresh media was added to cultures on day 3, and BMDM were cultured until day 6 before harvesting for further experiments. Alternatively, F4/80+ BMDMs were further enriched with magnetic particles (Miltenyi Biotec)

### Reagents

LPS and Necrostatin-1 were purchased from Sigma-Aldrich, and zVAD-FMK was purchased from Enzo Life Sciences. The following monoclonal antibodies were used in the studies: Pacific Blue-conjugated anti-CD3, anti-MHCII; APC-conjugated anti-CD4, anti-CD11c; APC-Cy7-conjugated anti-B220; PE-Cy7-conjugated anti-CD8, anti-CD11b; PE-conjugated anti-Ter119, anti-Ly6G, anti-F4/80, anti-CD86; FITC-conjugated anti-CD71, anti-Ly6C. All antibodies were purchased from BD Biosciences, eBiosciences, Biolegend, or University of California, San Francisco. For western blots, anti-FADD (M19) from Santa Cruz and anti-GAPDH from Cell Signaling were purchased.

### Western Blotting

Enriched BMDMs were lysed at 50 x 10^6^ cells/ml in cold NP-40 lysis buffer. 1% NP-40, 50 mM Tris-Cl, pH 7.6, 150 mM NaCl, 1 mM EDTA, 10% glycerol, sodium orthovanadate, sodium fluoride and supplemented with protease inhibitors (1 mM phenylmethylsulfonyl fluoride, 5 mM pepstatin, 0.01 mM Aprotinin, 0.01 mM Leupeptin, 1 mM benzamidine). Cleared lysate was boiled in SDS sample buffer, resolved on 10% SDS-PAGE gel, and probed with anti-FADD and anti-GAPDH antibodies.

### Cell Death Induction

BMDMs were plated in 24-well non-tissue culture-treated plates at 10^6^ cells/well in 1mL complete RPMI media. Samples were plated in duplicates. The cells were pre-treated with 10 μM zVAD-FMK and 30 μM Necrostatin-1 for 30 min and stimulated with 10 ng/ml LPS. Following 16–18 hr of stimulation, BMDMs were harvested in cold PBS and surface stained with anti-F4/80 and labeled with 7AAD. Samples were analyzed by flow cytometry.

### ELISA and Cytometric Bead Array

Blood was collected from tail vein or cardiac puncture. Flt3L (R&D Systems) was analyzed by ELISA kit. Inflammatory cytokines were quantitated using the mouse inflammation cytometric bead array kit (BD Biosciences). Samples were collected on an LSRII and analyzed with FCAP Array Software (BD Biosciences).

### Statistical Analysis

Statistical significance was calculated using paired Student’s t test. Mann-Whitney U test was used to compare survival curves. Statistical analysis was completed with GraphPad Prism. ***p<0.001 **p<0.01 *p<0.05

## Results

### mFADD^-/-^ Mice Exhibit Splenomegaly and Systemic Inflammation

**Fig 1:**
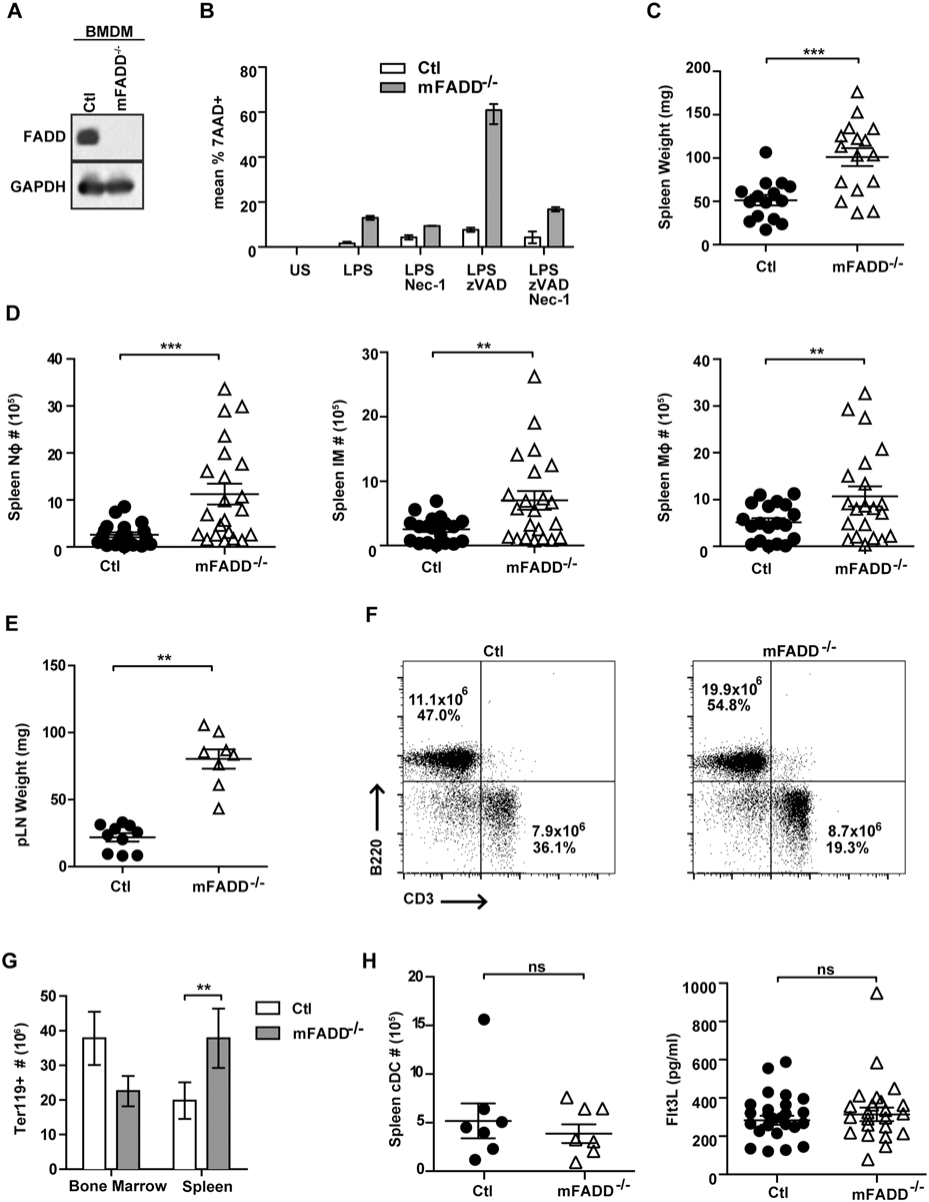
mFADD^-/-^ BMDMs are sensitive to death and mFADD^-/-^ mice exhibit expanded inflammatory cell populations. **A.** FADD protein expression was assessed in BMDMs by western blotting (Ctl, control). Western blot analysis with anti-GAPDH antibodies was performed for loading controls. **B.** Ctl (white bars) and mFADD^-/-^ (gray bars) BMDMs were stimulated with indicated treatments for 16–18 hrs. Cells were labeled with 7AAD and cell death calculated as 7ADD^+^. **C.** Spleen weights from Ctl or mFADD^-/-^ mice. **D.** Cell numbers of neutrophils (Nφ), inflammatory monocyte (IM) and macrophages (Mφ) from the spleens of Ctl or mFADD^-/-^ mice. **E.** Weights of peripheral lymph nodes (pLN) from Ctl or mFADD^-/-^ mice. **F.** Representative FACs plots of cells stained with CD3 and B220 to examine B cell and T cell populations from Ctl and mFADD^-/-^ mice. Numbers are indicative of total cell numbers and cell percentages. Data represent at least three separate experiments. **G.** The number of erythrocytes (Ter119+) in the bone marrow and spleen of Ctl (white bars; n = 6) of mFADD^-/-^ (gray bars; n = 7) mice. **H.** Splenic conventional dendritic cell numbers (cDC) from Ctl or mFADD^-/-^ mice (left panel). Flt3L concentrations from sera of Ctl or mFADD^-/-^ mice as determined by ELISA (right panel). For Figs 1C, 1D, 1E and 1H, each circle or triangle represents one mouse. ns denotes statistically not significant. ***p<0.001 **p<0.01 *p<0.05.

To examine the role of FADD in macrophages, we crossed *FADD* ^*fl/fl*^ mice to *LysM-Cre* mice to generate *LysM-Cre/FADD* ^*fl/fl*^ mice (mFADD^-/-^ mice). *LysM-Cre* mice express Cre under the control of the lysozyme promoter. When crossed to loxP-flanked target genes, deletion was reported in ~95–100% of macrophages and neutrophils [44]. No deletion was seen in T or B cells but partial deletion (16%) was seen in splenic DCs [44]. To confirm the extent of FADD deletion in macrophages, we generated bone marrow-derived macrophages (BMDM) and performed western blot analysis with FADD-specific antibodies. As seen in Fig 1A, FADD expression was undetectable in mFADD^-/-^ BMDM. We investigated the susceptibility of these macrophages to cell death and found that addition of LPS to mFADD^-/-^ BMDM but not the wild-type BMDM led to increased cell death (Fig 1B). This death was further enhanced by addition of zVAD-FMK, a general caspase inhibitor and could be rescued by Necrostatin-1 (Nec-1), a RIP1 kinase inhibitor [45] (Fig 1B). Interestingly, the rescue by Nec-1 was only partial, suggesting that some of the death was RIP1-independent but RIP3-dependent. In support of this, loss of RIP3 alleles completely abolished the LPS-induced necroptosis of mFADD^-/-^ macrophages (S1 Fig).

We analyzed 6–12 week-old mice and compared mFADD^-/-^ mice to their littermate controls (*FADD* ^*fl/fl*^ or *LysM-Cre/FADD* ^*fl/+*^). Spleen and lymph nodes were analyzed for various immune populations in a manner similar to that which was performed for dcFADD^-/-^ mice [34]. Interestingly, similar to dcFADD^-/-^ mice, mFADD^-/-^ mice suffer from splenomegaly (Fig 1C) and lymphadenopathy (Fig 1E) with increased neutrophils (Ly6C^lo^ CD11b^+^), inflammatory monocytes (Ly6C^hi^CD11b^+^), macrophages (CD11b^+^ F4/80^+^), B cells (B220^+^) and Ter119^+^ erythrocytes (Fig 1D, 1F, and 1G). The elevated number of macrophages is surprising given that *FADD*-deficient macrophages are sensitive to necroptosis. In addition, T cell composition and number appear to be the same between mFADD^-/-^ and littermate controls (Fig 1F and data not shown). We also analyzed the splenic DC number. As expected, they are similar to that of littermate controls for both splenic DCs (Fig 1H) and DCs in the gut-associated lymph nodes (see below). In support of this, we measured Flt3L levels in the serum by ELISA. Flt3L is required for DC differentiation and its levels inversely correlate with the number of DCs *in vivo* [46–48]. Both DC-less mice and dcFADD^-/-^ mice, which have no DCs and fewer DCs, respectively, exhibit elevated levels of Flt3L [34,46]. As shown in Fig 1H, the serum Flt3L levels are the same between mFADD^-/-^ mice and their littermate controls. Thus, this result confirms that mFADD^-/-^ mice contain normal numbers of DCs in their immune organs.

**Fig 2:**
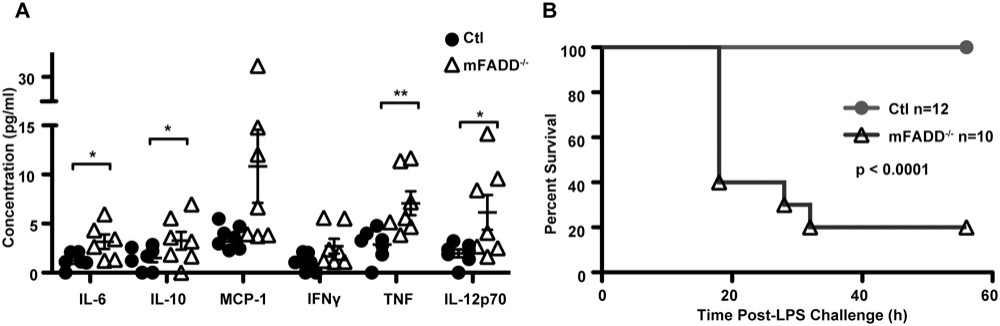
mFADD^-/-^ mice exhibit elevated inflammatory cytokines and succumb to LPS-induced endotoxic shock. **A.** Serum cytokine levels (pg/ml) were measured by flow cytometry using cytometric bead array (CBA) from Ctl (n = 7) or mFADD^-/-^ mice (n = 7). **p<0.01, *p<0.05. **B.** Ctl (n = 12) or mFADD^-/-^ (n = 10) mice were injected with 100μg LPS intraperitoneally, and survival was monitored.

To see if mFADD^-/-^ mice exhibit elevated inflammatory cytokines, we performed cytometric bead array on sera of 6–12 week-old mFADD^-/-^ mice. Similar to dcFADD^-/-^ mice, these mice exhibit a slight elevation of serum TNF (Fig 2A). However, unlike dcFADD^-/-^ mice, they also showed statistically significant increases in IL-6, IL-10, and IL-12. Moreover, injection with a low dose of LPS resulted in death of 80% of mFADD^-/-^ mice within 30 hrs (Fig 2B). In contrast, LPS did not cause any lethality to the littermate controls. Thus, mFADD^-/-^ mice exhibit systemic chronic inflammation and succumb to LPS-induced endotoxic shock similar to that of dcFADD^-/-^ mice.

### mFADD^-/-^ Systemic Inflammation is RIP3-Dependent

**Fig 3:**
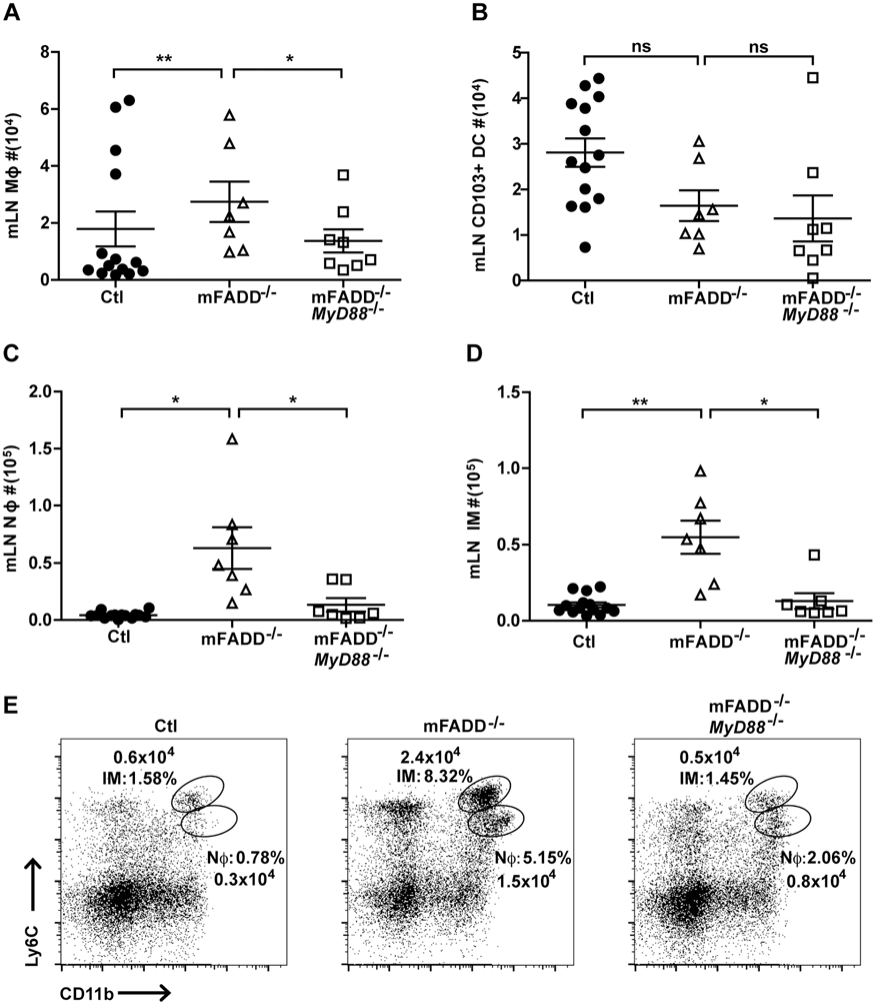
Increased cell numbers of inflammatory populations in the mesenteric lymph nodes of mFADD^-/-^ mice. A-D. Cell numbers of macrophages (Mφ), CD103+ DCs, neutrophils (Nφ), and inflammatory monocytes (IM) from the mesenteric lymph nodes (mLN) of control littermates (Ctl), mFADD^-/-^, and mFADD^-/-^MyD88^-/-^ mice. E. Representative flow cytometric analysis of inflammatory monocyte (IM: Ly6C^hi^CD11b+) and neutrophil (Nφ: Ly6C^lo^CD11b+) percentages and cell numbers from mLN. Each open or closed circle, triangle or box represents one mouse. ***p<0.001, **p<0.01, *p<0.05, ns: not significant.

In dcFADD^-/-^ mice, the lower number of DCs in GALT can be rescued by RIP3 deficiency. Moreover, systemic inflammation is resolved in these dcFADD^-/-^/*RIP3* ^-/-^ mice. These data suggest that gut microbiota stimulate DCs to die through necroptosis when apoptosis is blocked [34]. Necroptotic DCs then release inflammatory contents, which can be sensed by other cells, resulting in systemic inflammation. Subsequently, we assessed if *FADD*-deficient macrophages die through necroptosis after being stimulated by commensal microflora through their TLRs. Examination of F4/80 macrophages in mesenteric lymph nodes however, did not reveal a decrease in the number of macrophages (Fig 3A). As expected, no changes were observed in the numbers of CD103^+^ migratory DCs in mesenteric lymph nodes (mLN),which is consistent with the lack of changes in Flt3L serum levels (Figs 3B and 1G). Thus, the systemic inflammation of mFADD^-/-^ is unlikely due to any possible effects from their DCs. As seen in the spleen, neutrophil and inflammatory monocyte numbers were still increased in the mesenteric lymph nodes of mFADD^-/-^ mice (Fig 3C, 3D, and 3E).

**Fig 4:**
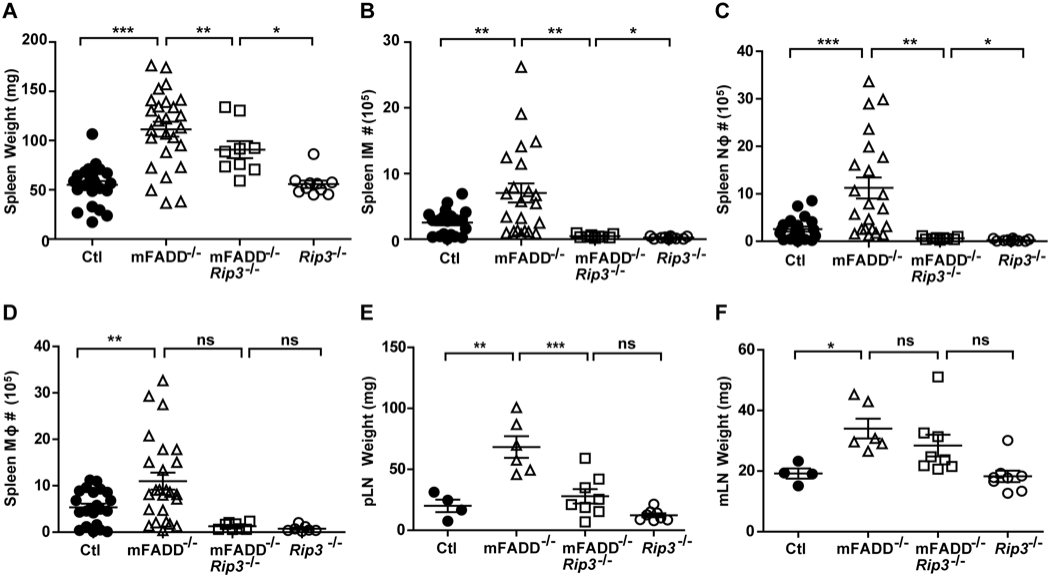
Loss of RIP3 rescues systemic inflammation found in mFADD^-/-^ mice. **A.** Spleen weights of mFADD^-/-^ mice, their littermate controls (Ctl) and age-matched mFADD^-/-^RIP3^-/-^ mice and their RIP3^-/-^ littermates. **B-D.** Cell numbers of inflammatory monocytes (IM), neutrophils (Nφ), and macrophages (Mφ) from the spleens of Ctl, mFADD^-/-^, mFADD^-/-^RIP3^-/-^, or RIP3^-/-^ mice. **E.** Combined weights of axillary, brachial, and inguinal lymph nodes (pLN) from indicated mice. **F.** Weights of mesenteric lymph nodes (mLN) of indicated mice. Each open or closed circle, triangle or box represents one mouse. ***p<0.001, **p<0.01, *p<0.05, ns: not significant.

To examine the role of RIP3, we crossed mFADD^-/-^ mice to *RIP3* ^*-/-*^ mice. As shown in Fig 4, analysis of mFADD^-/-^/*RIP3* ^*-/-*^ and *RIP3* ^*-/-*^ littermates in comparison to mFADD^-/-^ and wild-type controls indicate that the mFADD^-/-^ phenotypes are partially rescued in mFADD^-/-^/*RIP3* ^*-/-*^ mice. Although there is a slight increase in spleen weight, neutrophils, and inflammatory monocytes in mFADD^-/-^/*RIP3* ^*-/-*^ mice when compared to *RIP3* ^*-/-*^ mice, the numbers were reduced as compared to mFADD^-/-^ mice (Fig 4A, 4B and 4C). Furthermore, the number of splenic macrophages as well as peripheral lymph node and mesenteric lymph node weights in mFADD^-/-^/*RIP3* ^*-/-*^ mice were similar to that found in *RIP3* ^*-/-*^ mice (Fig 4D, 4E, and 4F). The most well characterized function of RIP3 is its role in necroptosis induction, the subsequent promotion of inflammation is thought to be a secondary event due to the release of damage-associated molecular patterns (DAMPs) by necrotic cells [34,49]. However it has recently been appreciated that RIP3 can also function directly in promoting inflammation through production of inflammatory cytokines [38–40,42]. Thus, RIP3-dependent inflammatory activity, whether indirectly through necroptosis or directly through promotion of inflammatory cytokines, is responsible for the systemic inflammatory phenotype found in mFADD^-/-^ mice.

### MyD88-Dependent Signaling Is Crucial for mFADD^-/-^ Inflammation

**Fig 5:**
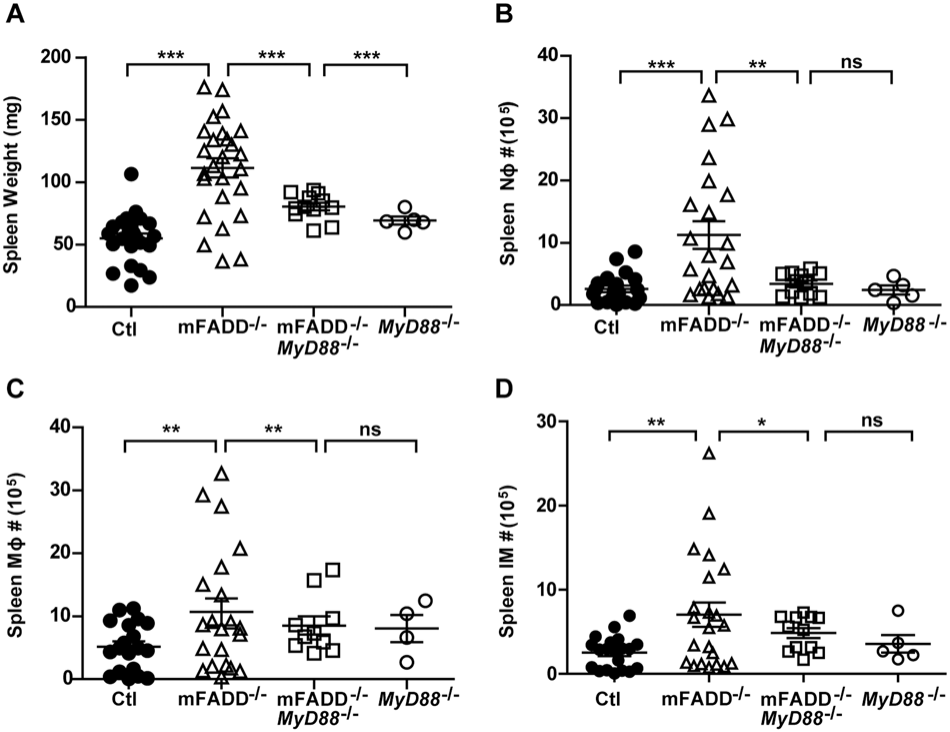
MyD88-dependent signaling is crucial for systemic inflammation observed in mFADD^-/-^ mice. **A.** Spleen weights of mFADD^-/-^MyD88^-/-^ mice, their MyD88^-/-^ littermates and age-matched mFADD^-/-^ and their wild-type littermate controls (Ctl). **B-D.** Cell numbers of neutrophils (Nφ), macrophages (Mφ), and inflammatory monocytes (IM) in the spleens of mFADD^-/-^MyD88^-/-^ mice as compared to their MyD88^-/-^ littermates, aged-matched mFADD^-/-^ mice and wild-type controls (Ctl). Each open or closed circle, triangle or box represents one mouse. ***p<0.001, **p<0.01, *p<0.05.

We have previously shown that MyD88, an adapter protein essential for most TLR signaling, is required for the systemic inflammatory phenotypes of dcFADD^-/-^ mice [34]. To assess the requirement of MyD88 in *FADD*-deficient macrophage-induced inflammation, we similarly crossed mFADD^-/-^ mice to *MyD88* ^*-/-*^ mice. Analysis was then carried out for mFADD^-/-^/*MyD88* ^*-/-*^ mice for comparison to *MyD88* ^*-/-*^ littermates. In some cases, we were also able to obtain mFADD^-/-^ littermates for our analysis. We found that loss of MyD88 rescued the inflammatory phenotype in mFADD^-/-^ mice. A decrease in spleen weight and normal numbers of neutrophils, inflammatory monocytes and macrophages were seen in mFADD^-/-^/*MyD88* ^*-/-*^ mice (Fig 5). Likewise, loss of MyD88 rescued cell numbers seen in the mesenteric lymph nodes (Fig 3). These data suggest that aberration in cell death machinery, whether in macrophages or DCs, results in MyD88-driven inflammation [34].

## Discussion

In this paper, we showed that FADD is not required for normal macrophage development or proliferation but that the loss of FADD results in macrophage sensitivity to LPS-induced necroptosis and RIP3-dependent inflammation. This is similar to TLR-stimulated *FADD*-deficient DCs [34]. TLR3 and TLR4 in macrophages/DCs can presumably activate necroptosis through association of RIP3 with the adapter protein TRIF [35,36]. We have previously shown that *FADD*-deficient DCs can be stimulated to undergo necroptosis through MyD88 as well, although the molecular mechanism of MyD88-dependent death in DCs is not clear. In bone marrow-derived macrophages, MyD88-dependent necroptosis *in vitro* was reported to occur through a TNF-dependent mechanism [36]. However, we found that addition of a TNF neutralizing antibody did not rescue LPS induced death of mFADD^-/-^ BMDM (S1 Fig). Nevertheless, it is possible that other innate immune stimuli may result in TNF-dependent necroptosis and therefore contribute to the inflammation observed. Alternatively, MyD88 may directly activate RIP3 *in vivo* through a novel mechanism without going through the TNF pathway.

Here we report that mFADD^-/-^ inflammatory phenotypes disappear on a *MyD88*-deficient background. It is unlikely that the rescue is due to a complete absence of necroptotic macrophages/neutrophils as TRIF can still provide signals to trigger cell death. Consistent with this, DC-specific loss of MyD88 only partially rescues the dcFADD^-/-^ phenotypes [34]. Although we didn’t generate macrophage-specific *MyD88*-deficient mice, we expect the results to be similar to that of dcFADD^-/-^/dcMyD88^-/-^ mice. In dcFADD^-/-^ mice, the inflammatory phenotype is also rescued by antibiotics administration. Together, these data are consistent with the notion that innate immune cells are primed continuously through MyD88 signaling, and this is crucial for their ability to respond to danger signals including those released by necrotic cells.

Unlike dcFADD^-/-^ mice where a significant reduction of DC number in GALT was detected, the number of macrophages in mFADD^-/-^ mice is surprisingly elevated instead of decreased. Since the observed phenotypes in mFADD^-/-^ mice are similar to that of dcFADD^-/-^ mice, we considered the possibility that leakiness of the lysozyme-driven Cre in DCs could result in a significant number of necroptotic FADD-deficient DCs in mFADD^-/-^ mice. Although we did find the number of CD103^+^ DCs in mesenteric lymph nodes of mFADD^-/-^ mice to be mildly decreased, the number was not statistically different from their littermate controls (Fig 3B). Moreover, the serum Flt3L levels were completely normal in mFADD^-/-^ mice. Flt3L levels are an excellent indicator of DC homeostasis in mice as they inversely correlate with DC number as shown in our previous analysis of dcFADD^-/-^ mice and in DC-less mice [34,46]. Thus, our data indicate that it is unlikely that necroptotic DCs are responsible for the mFADD^-/-^ phenotypes.

Our observation of increased macrophage cell numbers appears to disagree with necroptosis being the sole or major contributor to mFADD^-/-^ systemic inflammation. Recently, it was reported that RIP3 may promote inflammation independent of its role in necroptosis [38–40,42]. Our data on increased macrophage cell numbers appears consistent with this direct RIP3-inflammatory role. It has been reported that macrophages and dendritic cells deficient for caspase-8 or cIAP1, cIAP2, and XIAP can promote RIP3-dependent production of IL-1β in response to LPS [41,42]. Furthermore, LPS-induced IL-1β maturation in dendritic cells was shown to consist of two RIP3-dependent pathways involving activation of caspase-1 or caspase-8 [38]. We found that loss of RIP3 from mFADD^-/-^ mice rescued the inflammatory phenotype of our mice (Fig 4). Thus, although *RIP3*-deficient macrophages have been reported to have no defect in NF-κB activation or pro-inflammatory cytokine production in response to TLR or TNF stimulation, it is possible that other innate immune stimuli may activate non-necroptotic RIP3 inflammatory activity in mFADD^-/-^ mice [39,50].

Alternatively, it is plausible that a small population of *FADD*-deficient macrophages that are stimulated to die release inflammatory DAMPs, which results in proliferation of the rest of the macrophage population. Increased numbers of macrophages in response to necroptotic DCs was one of the phenotypes observed in the dcFADD^-/-^ mice [34]. In addition, we observed that LPS stimulation alone caused a small increase in cell death of *FADD*-deficient macrophages (Fig 1). Consequently, the inflammatory contents released by these dying macrophages in the mFADD^-/-^ mice may also stimulate other immune cells, leading to their activation and contributing to chronic inflammation. Given the emerging data on RIP3’s role as both a direct and indirect contributor to inflammation, it is likely that rescue of systemic inflammation in mFADD^-/-^ *RIP3* ^-/-^ mice is due not only to loss of macrophage necroptosis but also loss of RIP3-dependent inflammatory activity. This suggests that in vivo, FADD may play an important role in limiting RIP3 driven inflammatory activity, whether it be through necroptosis or other inflammatory pathways. As many of the same proteins identified in necroptosis induction have also been implicated in RIP3’s non-necroptotic inflammatory activity, there is a need for additional studies to fully evaluate the contribution and activation of these disparate functions [42,51,52].

In conclusion, the data presented here as well as that from dcFADD^-/-^ mice demonstrate the dynamic relationship between immune cells and the microbiota. They support the notion that these innate immune cells are important sentinels of the immune system, poised to respond to aberrations in cell death signaling and DAMPs.

## Supporting Information

S1 Fig Characterization of cell death in FADD-deficient macrophages. **A.** Cell death is rescued in mFADD^-/-^ *RIP3* ^-/-^ BMDM after LPS treatment. BMDM from indicated genotypes were not treated (US) or stimulated with a different combination of zVAD, Nec-1, and LPS. **B.** LPS induced death of mFADD^-/-^ BMDM is not rescued by TNF neutralization antibody. Addition of a TNF neutralizing antibody (5μg/ml) was unable to rescue LPS induced cell death of mFADD^-/-^ BMDM (gray bars).
(TIF)
