# Supplementary figures and images for: Deletion of FADD in Macrophages and Granulocytes Results in RIP3- and MyD88-Dependent Systemic Inflammation
Source: PLoS One. 2015 Apr 13;10(4):e0124391. doi: 10.1371/journal.pone.0124391 (PMC4395384; doi:10.1371/journal.pone.0124391)

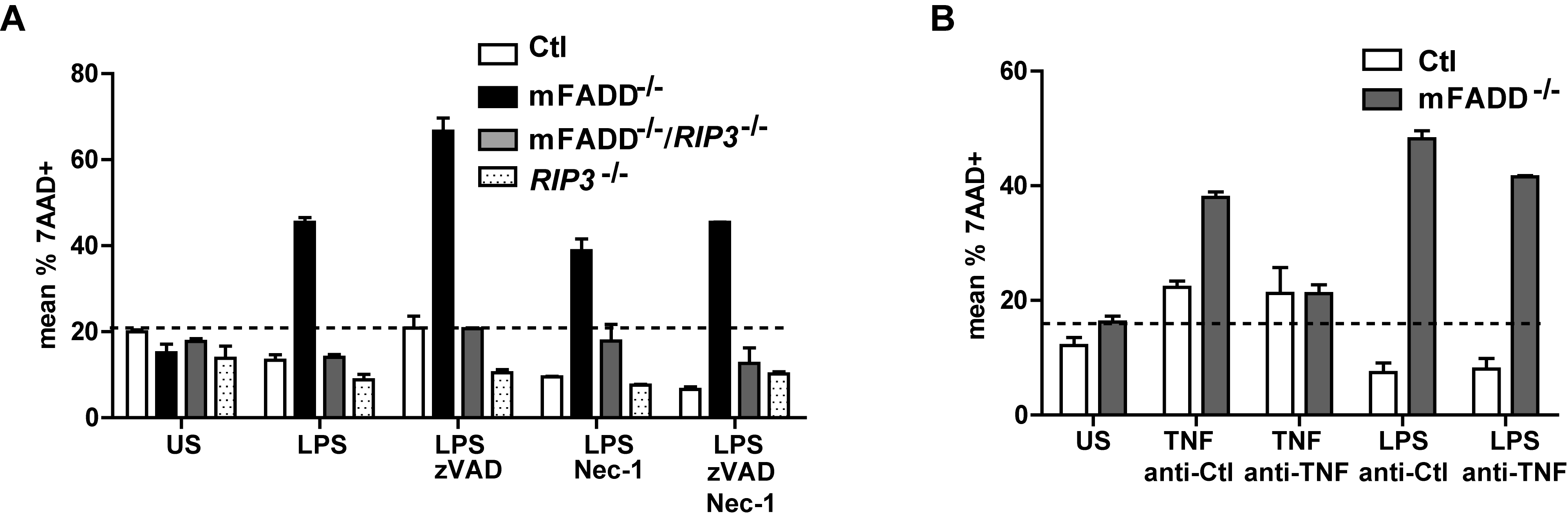

Supplement: S1 Fig — A. Cell death is rescued in mFADD-/- RIP3 -/- BMDM after LPS treatment. BMDM from indicated genotypes were not treated (US) or stimulated with a different combination of zVAD, Nec-1, and LPS. B. LPS induced death of mFADD-/- BMDM is not rescued by TNF neutralization antibody. Addition of a TNF neutralizing antibody (5μg/ml) was unable to rescue LPS induced cell death of mFADD-/- BMDM (gray bars). (TIF) [file pone.0124391.s001.tif]
